# Intrinsic innervation of the ovary and its variations in the rat senescence process

**DOI:** 10.1007/s10735-022-10069-7

**Published:** 2022-02-25

**Authors:** Juan M Bravo-Benitez, Yolanda Cruz, Rosa A Lucio, Berenice Venegas, Alfonso Díaz, Leticia Morales-Ledesma, Roberto Domínguez, Carolina Morán

**Affiliations:** 1 Doctorado en Ciencias Biológicas, Universidad Autónoma de Tlaxcala, Tlaxcala, Mexico; 2 Centro Tlaxcala de Biología de la Conducta, Universidad Autónoma de Tlaxcala, Tlaxcala, Mexico; 3 Facultad de Ciencias Biológicas, Benemérita Universidad Autónoma de Puebla, Puebla, Mexico; 4 Departamento de Farmacia, Facultad de Ciencias Químicas, Benemérita Universidad Autónoma de Puebla, Puebla, Mexico; 5 Biology of Reproduction Research Unit, Physiology of Reproduction Laboratory, Facultad de Estudios Superiores Zaragoza, Universidad Nacional Autónoma de México, CDMX, Mexico; 6 Centro de Investigación en Fisicoquímica de Materiales, Instituto de Ciencias, Benemérita Universidad Autónoma de Puebla, Puebla, Mexico

**Keywords:** Ovarian innervation, Senescent rat, Intrinsic neurons, Ovarian function

## Abstract

Ovarian functions decrease with perimenopause. The ovary has extrinsic innervation, but the neural influence on ovarian functions and dysfunction is not well-studied. The present study aimed to biochemically and morphometrically characterize the intrinsic neurons in ovaries from young adult, middle-aged, and senescent Long Evans CII-ZV rats (3, 12, and 15 months old, respectively). Ovaries were extracted from four rats of each age group (n = 12 total), cryopreserved, and processed for immunofluorescence studies with the primary NeuN/β-tubulin and NeuN/tyrosine hydroxylase (TH) antibodies. The soma area and number of intrinsic neurons in the ovarian stroma, surrounding follicles, corpus luteum, or cyst were evaluated. The intrinsic neurons were grouped in cluster-like shapes in ovarian structures. In senescent rats, the intrinsic neurons were mainly localized in the ovarian stroma and around the cysts. The number of neurons was lower in senescent rats than in young adult rats (p < 0.05), but the soma size was larger than in young adult rats. Immunoreactivity to TH indicated the presence of noradrenergic neurons in the ovary with the same characteristics as NeuN/β-tubulin, which indicates that they are part of the same neuronal group. Taken together, the findings indicate that the intrinsic neurons may be related to the loss of ovarian functions associated with aging.

## Introduction

In mammals in the mid-life stage, an exponential decrease in the number of ovarian follicles correlates with the beginning of female subfertility, a process known as reproductive senescence. Once this event occurs, there is a decrease in viable oocytes. In general, when the pool of follicles in a woman’s ovaries is < 1000, the hormonal feedback with the hypothalamus cannot be sustained, which leads to menopause (Cruz et al. 2017; te Velde et al. 1998). In Sprague Dawley and Wistar rats, a decrease in the number of follicles and modification of the estrous cycle has been observed at 10 months of age (Acuna et al. 2009; Peng and Huang 1972). The decrease in follicles continues until 12 months old (middle-aged), then the rats experience subfertile and infertile periods as they grow older (senescent) (Acuna et al. 2009; Chavez-Genaro et al. 2007).

The configuration of the ovarian tissue undergoes structural and functional change depending on the age of the animal. Changes include proliferation, differentiation, and cellular death, (e.g., follicle growth, ovulation, forming of the corpus luteum [CL], cysts, and ovarian stroma [OS]). As the animal ages, there are modifications in the ovarian configuration; mainly, cysts begin to appear when rats are 6 months old and, by the age of 14 months (senescent rat), large cysts increase in number and occupy most of the ovarian cortex (Acuna et al. 2009).

The ovarian extrinsic innervation involves autonomic and sensorial components that reach the ovaries through the superior ovarian nerve and ovarian plexus nerve (Aguado 2002; Baljet and Drukker 1979; Doganay et al. 2010; Pastelin et al. 2017). In some species, the nerve fibers reach the primordial and in-development follicles but do not penetrate the basal lamina to the granulose cells or CL (D'Albora and Barcia 1996; D'Albora et al. 2000). For example, in the female pig, the parasympathetic intraovarian nerve fibers surround the preantral and antral follicles, the CL, blood vessels, and OS. Such fibers release acetylcholine (Ach) and co-transmitters, including nitric oxide, vasoactive intestinal polypeptide (VIP), somatostatin (SOM), substance P, and galanin (Jana et al. 2018). In pathological conditions (hyperestrogenism), the quantity of parasympathetic nerve fibers in the adult pig ovary decreases in the inner part of the ovary, altering the cholinergic innervation pattern, neuronal isoform of nitric oxide synthase (nNOS), and VIP, which in turn modifies its functions (Jana et al. 2018).

Other studies have described an association between a loss in the number of fertile follicles and an increasing concentration of norepinephrine (NE) in the rat ovary (Chavez-Genaro et al. 2007). Thus, an experimental increase in the ovarian sympathetic tone of 10-month-old animals increases catecholamine levels, which correlates with cyst formation, along with follicular degeneration. In the ovaries of 14-month-old animals, the number of cysts was significantly high (Acuna et al. 2009). However, in the celiac ganglia (main neuronal relay between the central nervous system and ovaries), the levels of NE do not increase during the senescent period, but decrease with age. Therefore, the increase in NE may occur via intrinsic innervation, which has not been studied in senescent stages (Acuna et al. 2009; Banerjee et al. 2014; Chavez-Genaro et al. 2007; Dumesic and Richards 2013; Mayerhofer et al. 1998; Venegas-Meneses et al. 2015).

Intrinsic innervation and its participation in functioning has been demonstrated in diverse organs (Anetsberger et al. 2018; Hao et al. 2020). The intrinsic innervation of the ovary has been described in several species (Gilbert 1969; Madekurozwa 2008). In pigs, nerve structures, such as neuronal cells and nerve fibers, have been identified inside the ovary (Jana et al. 2015). In humans, neuronal intraovarian cells have also been described using anti-human p75-NGFR, with reports of neural cells with a prominent nucleus around the follicles, CL, medulla, and ovarian cortex (Anesetti et al. 2001), but without penetrating them. In rodents, neurons in the ovaries of Wistar rats have been identified using morphogenic markers (NeuN), as well as nerve growth factor (NGF) and tyrosine hydroxylase (TH) for differentiated neurons. These neurons have a multipolar soma and are localized in the ovarian medulla during neonatal and young adult stages. However, these neurons have not been found in Sprague–Dawley rats (D'Albora and Barcia 1996; D'Albora et al. 2000).

The aim of the present study was to locate and biochemically/morphometrically characterize the intrinsic neurons in the ovaries of young adult, middle-aged, and senescent Long Evans CII-ZV rats. The antibodies used in this study were chosen due to their specificity and ability to recognize neuronal bodies in the central or peripheral nervous systems. NeuN has been found in the nuclei of neurons of the central nervous system and in the cytoplasm of peripheral neurons in the enteric system (Dredge and Jensen 2011; Duan et al. 2016; Gusel'nikova and Korzhevskiy 2015; Kim et al. 2009; Lind et al. 2005; Mullen et al. 1992; Van Nassauw et al. 2005; Weyer and Schilling 2003). Considering that a reduction in NeuN has been observed in the spinal cord of senescent rats compared to young rats (Portiansky et al. 2006), we decided to use a second neuronal marker to identify intrinsic neurons in the ovary, type-III β-tubulin (Almeida-Souza et al. 2011; Baas et al. 2016; Dredge and Jensen 2011; Katsetos et al. 2003; Kim et al. 2009; Lind et al. 2005; Mariani et al. 2015; Portiansky et al. 2006; Weyer and Schilling 2003). The synthesis of this marker increases with age; NeuN loses immunoreactivity, whereas the expression of β-tubulin increases (Almeida-Souza et al. 2011; Baas et al. 2016; Jiang and Oblinger 1992; Korzhevskii et al. 2011).

## Materials and methods

### Animals

The animals were provided by Bioterio Claude Bernard, BUAP. The protocol was approved by the Committee to Use and Care for the Laboratory Animals of Benemérita Universidad Autónoma de Puebla (Of. VIEP 1040/2019). All procedures described in this work were carried out in accordance with the guidelines for the use and care of laboratory animals by the Mexican Committee for Animal Care (NOM-062-ZO-1999). Every effort was made to minimize the number of animals in each experimental group and to ensure minimal discomfort and pain. Twelve adult female CII-ZV rats were used. They were kept in controlled conditions (14 h light/10 h dark, lights on from 05:00 to 19:00 h) with free access to water and food.

### Experimental design

The females were assigned to the following groups: young adult rats aged 3–5 months (3 M, n = 4), middle-aged rats aged 12 months (12 M, n = 4), and senescent rats aged 15 months (15 M, n = 4). Both ovaries from each animal were analyzed (i.e., eight ovaries per group). In the 3 M group, only those in estrous were used (rats with three consecutive 4-day estrous cycles, daily vaginal smears were taken between 09:00 and 10:00 h). In the 12 M group, rats were used only if vaginal cytology showed continuous estrous (regular estrus cycle and ovulation ceased, vaginal cytology indicated desquamation cells in vagina). In the 15 M group, rats were used only if vaginal smears indicated continuous anestrus (persistent leukocytes).

### Ovary dissection

Animals from each group were deeply anesthetized with pentobarbital between 09:00 and 11:00 h (80 mg/kg weight, i.p.). The animals were then perfused with 200 mL of Hartmann solution and the ovaries dissected during the necropsy. The ovaries were placed in fixative (Carnoy) for 24 h, rinsed with Hartmann solution, and kept in solution with 30% sucrose until immunofluorescence processing.

### Immunofluorescence

The preserved ovaries were cut into 10-µm sections using a cryostat (THERMO Shandon cryotome E) at − 25 °C. The sections were defrosted at room temperature and treated for 90 min in 6% fetal bovine serum (FBS; Cat. 26140079, Gibco Thermo Scientific) and 0.1% Tween 20 (Sigma Chemical Co., St. Louis, Mo. USA). Next, they were incubated with the primary antibody for 72 h at 4 °C. Polyclonal antibody anti-rabbit β-tubulin (1:500, Cat. 2128 Cell Signaling) or anti-rabbit tyrosine hydroxylase (1:500, Cat. AB 152 Merck Millipore) and anti-mouse NeuN (1:500, Cat. MAB 377 Merck Millipore) antibodies were used. After incubation with the primary antibody, the tissues were washed with 0.5 M Tris–EDTA buffer (BTE) for 1 min. They were incubated with the secondary antibodies, anti-rabbit coupled with rhodamine (1:500, Jackson Immuno Research Laboratories, Inc.) and anti-mouse coupled with fluorescein (1:500, Jackson Immuno Research Laboratories, Inc.), for 24 h. At the end of this incubation, the tissues were washed with BTE for 1 min. DAPI-Vecta-Shield was added to the tissues (Vector Laboratories, Burlingame, CA, USA) for observation under the microscope.

For negative controls, the primary antibody was substituted with 6% FBS and BTE. Observations and analyses of the immunoreactivity of the antibodies in the ovarian follicles were performed using a fluorescence microscope (Olympus BX41) equipped with an Evolution VF Digital Camera (Media Cybernetics, Inc., USA).

### Statistical analysis

Immunoreactive neurons to primary antibodies were counted and the area of the soma determined using Image Pro-premier 9.2 software (Media Cybernetics, Inc., Rockville, USA). To obtain the soma area of the intrinsic neurons, the immunoreactive cells, including well-defined nuclei, were selected and delineated manually in microphotographs and the area of the intrinsic neurons measured by the software. To define the number of neurons around each follicle or cyst, the Block method for estimation of cell populations (Block, 1951, 1952) was used as a correction factor. From the total number of slides, 1 in 5 was recovered and selected for the following groups: NeuN/β-tubulin; NeuN/TH; negative control for NeuN/β-tubulin; negative control for NeuN/TH; and H&E. Significant differences among experimental groups were determined by ANOVA followed by a Tukey test for nonparametric data (Graphpad Prism 8.1).

## Results

**Fig. 1:**
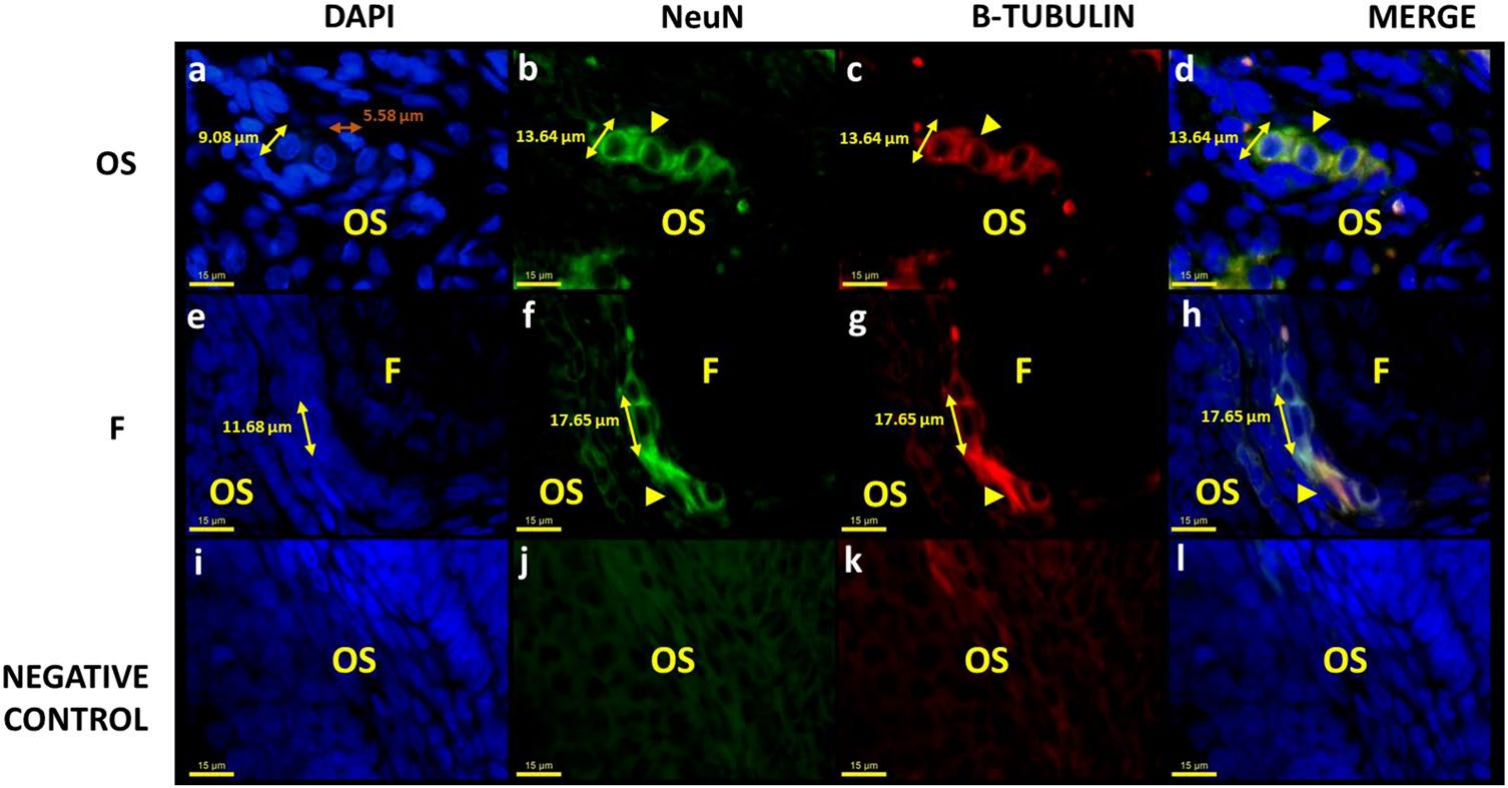
Microphotographs show a cluster of neurons in the ovarian stroma (OS) and follicles (F) of young adult (3 M) female rats. **a**, **e** The nuclei are labeled with DAPI. **b**, **f** NeuN. **c**, **g** β-Tubulin. **d**, **h** Co-localization of NeuN and β-tubulin. The yellow head arrows show intrinsic neurons of the ovaries, in the theca layer of the follicle. The yellow double line arrow **a** show the average nucleus of the intrinsic neuron, twice the size of other ovarian cells (orange double line arrow). **i**, **j**, **l** Negative control

**Fig. 2:**
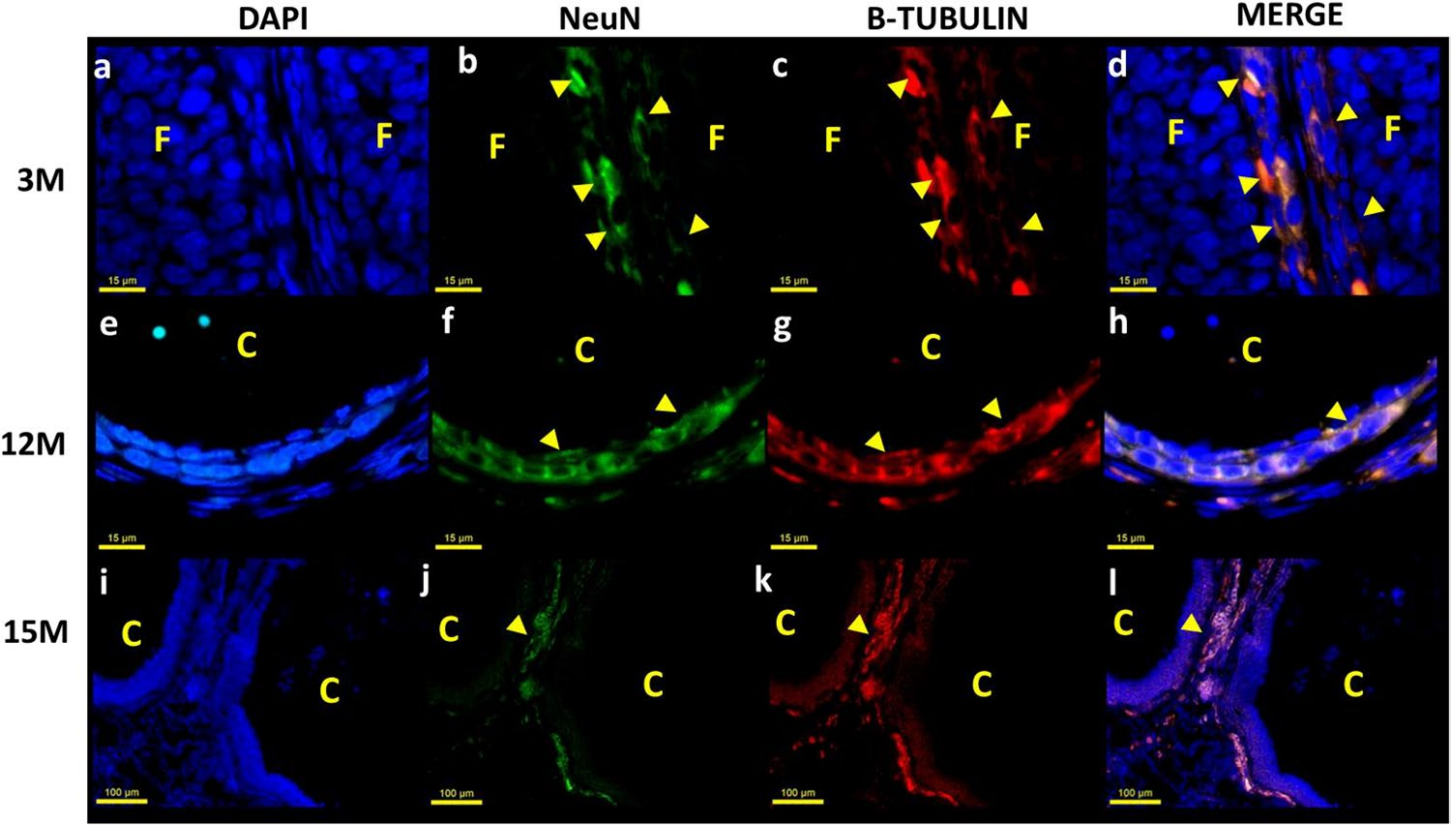
Immunofluorescent staining was used to localize intrinsic neurons in the ovary using NeuN (green) and β-tubulin (red). **a**, **e**, **i** Nuclei were labeled with DAPI. **b**, **c** Distribution of the intrinsic neurons around the follicles (F) of the ovaries in a young adult rat (3 M). **f**, **g** Around a cyst (C) of the middle-aged rat (12 M) (× 60). **j**, **k** Around a cyst in a senescent rat (15 M). **d**, **h**, **l** Co-localization of NeuN and β-tubulin. The yellow head arrows show intrinsic neurons of the ovaries

**Fig. 3:**
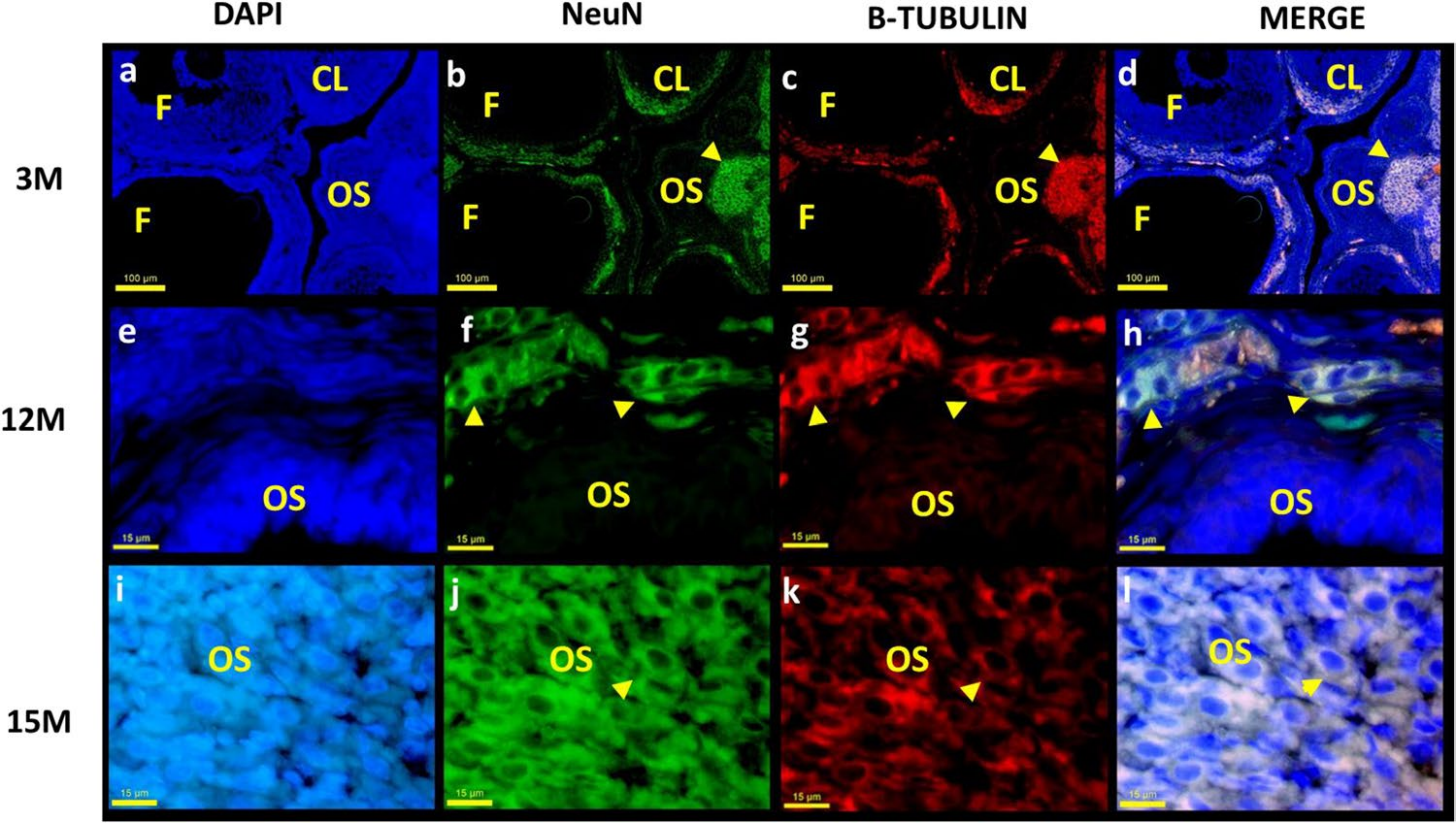
The top microphotographs are of ovaries from young adult rat (3 M), middle-aged rat (12 M), and senescent rat (15 M). Immunofluorescent staining was performed with mouse anti-NeuN (green) and rabbit anti-β-tubulin (red) antibodies, showing intrinsic neurons in the ovarian stroma (OS), corpus luteum (CL), and follicles (F). **a**, **e**, **i** Nuclei were labeled with DAPI. **b**, **f**, **j** Neu-N. c,g,k: β-Tubulin. **d**, **h**, **l** Co-localization of NeuN and β-tubulin. The yellow arrows show intrinsic neurons of the ovaries

Intrinsic neurons were observed in the ovaries of all rats (Figs. 1, 2 and 3). These neurons showed different morphological characteristics from other ovarian cells, particularly a prominent, well-defined nucleus (average nucleus diameter of all ages 9.08 µm; Fig. 1), approximately twice the size of other ovarian cells (average nucleus diameter 5.58 µm). Although without specific staining it would be difficult to distinguish neurons by nucleus size, because the wide cellular diversity in the ovary.

In the 3 M group, the neurons were localized around the follicles of different development stages, the CL, cysts, and the OS. All neurons were observed to be in a cluster-like shape (Fig. 1) similar to small nerve ganglia. Figure 2a–d shows two contiguous follicles with parallel theca, the latter containing neurons. The ovaries from the 12 M group had atretic follicles and CL in regression, as well as ovarian cysts. The neurons in this stage were localized around the follicular cyst (Fig. 2), with a tendency to concentrate in the middle region of the ovary.

Follicles in different developmental stages and CL occupy the ovarian cortex area in young animals, with intrinsic neurons around them (Fig. 3a–d). In the ovaries of the 12 M and 15 M groups, the neurons were observed in the OS (Fig. 3i–l) and surrounding cystic structures (Fig. 3e–h).

**Fig. 4:**
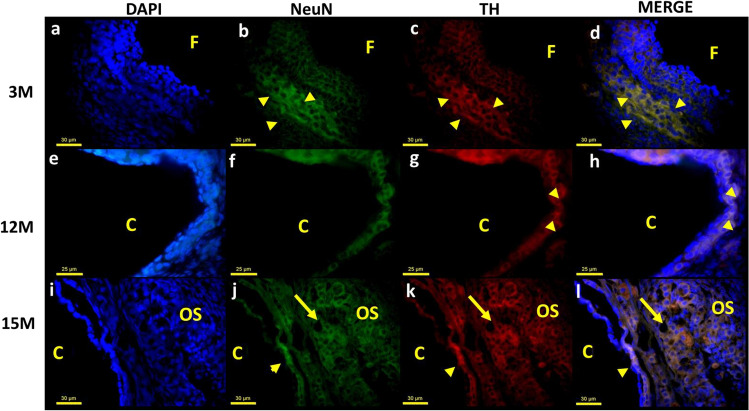
The top microphotographs are of ovaries from young adult rat (3 M), middle-aged rat (12 M), senescent rat (15 M). Inmunofluorescent staining was performed with mouse anti-NeuN (green) and rabbit anti-TH (red) antibodies. **b**, **c**, **d** show noradrenergic neurons in the theca layer of follicle. **f**, **g**, **h** show noradrenergic neurons around the cyst (C). **j**, **k**, **l** show noradrenergic neurons in ovarian stroma (OS) and around the cyst. The yellow head arrows show noradrenergic neurons around the follicle or cyst, and the yellow arrows show noradrenergic neurons on the ovarian stroma in the Senescent rat

In the 3 M group, the immunoreactive neurons to NeuN/TH localized around the follicles, as described for NeuN/β-tubulin positive neurons (Fig. 4a–d). TH immunoreactivity around the cyst was more abundant in the 12 M group (Fig. 4e–h, arrowhead) than in senescent animals (Fig. 4i–l, arrowhead). In the senescent rats, the TH-positive neurons (arrow) around the ovarian cysts were lower in contrast to the neurons in the OS (arrows). This indicates a lower number of neurons with ability to synthetized catechalamines.

**Fig. 5:**
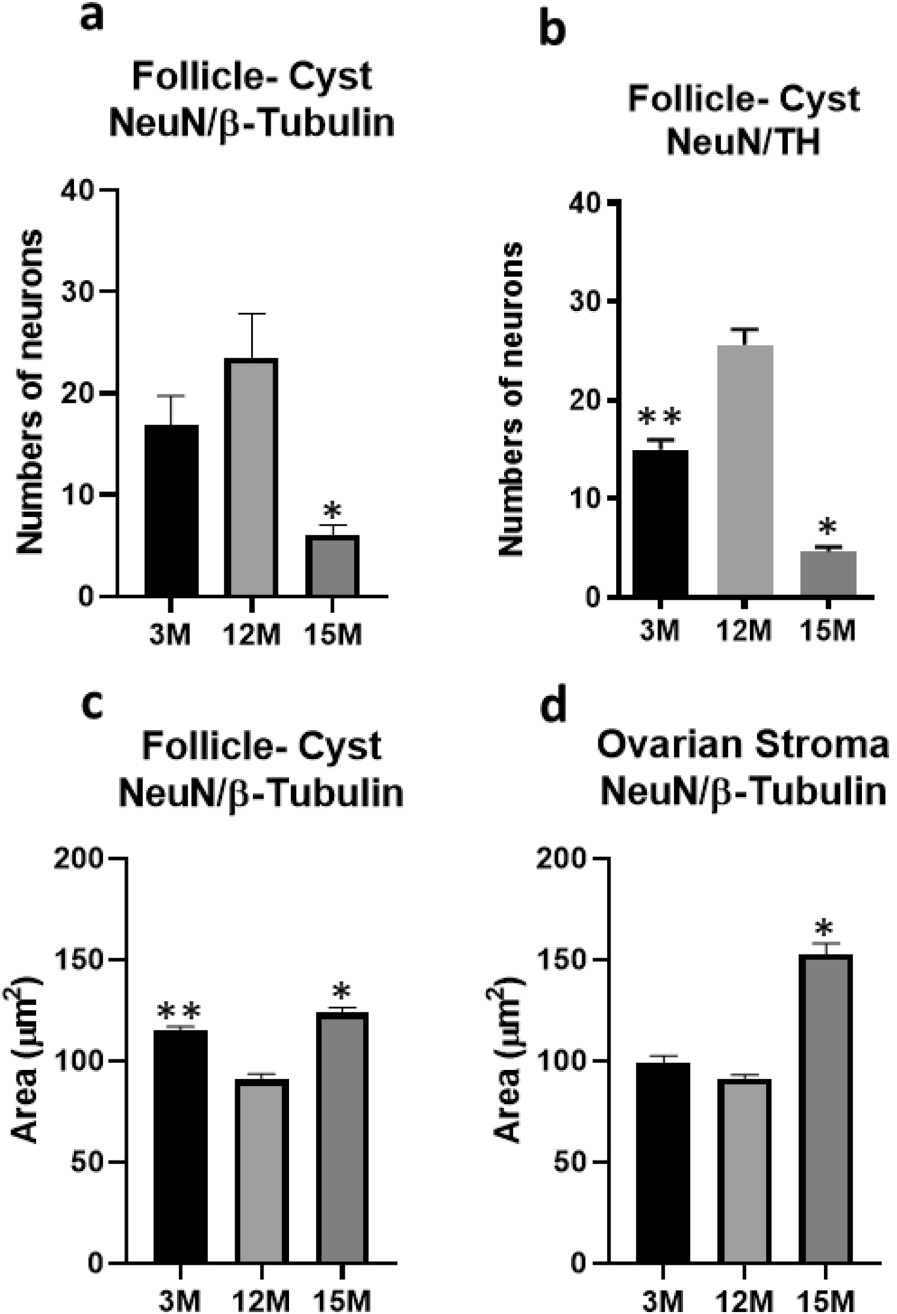
**a** Median number of intrinsic neurons (NeuN/β-tubulin) in ovaries from young adult rat (3 M), middle-aged rat (12 M), and senescent (15 M) CII-ZV rats. **b** Median number of intrinsic neurons (NeuN/TH) in ovaries **c** Median area of the intrinsic neurons (NeuN/β-tubulin) from ovarian follicles and cysts. **d** Median area of the intrinsic neurons (NeuN/β-tubulin) from ovaries in the ovarian stroma. *P < 0.05 vs. 3 M and 12 M, **P < 0.05 vs 12 M and 15 M; ANOVA followed by Tukey. Error bars indicate SEM

The total number of intrinsic neurons localized in the ovaries, around the follicles and cysts, varied throughout the life of the animals (Fig. 5a). In the senescent group, there were no more follicles or CL; however, there were neurons around the cysts. Regarding the number of neurons around the CL, there was no significant difference between the 3 M and 12 M groups (10.7 ± 1.6 vs. 6.6 ± 0.06, respectively).

In the OS, there were diverse and abundant intrinsic neurons, with no differences between age groups. However, comparing the number of neurons around follicles (3 M or 12 M) or cysts (15 M) between the groups showed that senescent rats had fewer neurons than middle-aged rats (Fig. 5a), these data indicates that elevated intrinsic innervation in ovaries is relevant in the loss of ovulatory capacity process (12 M) and from this stage the innervation it’s significantly reduced by aging (15 M).

Nevertheless, the area of the neurons was larger in the senescent rats than in the other groups (Fig. 5c, p < 0.05). The number of NE-neurons was different in the groups, with a peak in the middle-aged rats (Fig. 5b). The area of the NE-neurons surrounding the follicles and ovarian cysts was also different between the groups, as it increased proportionally with age. An age-dependent increase in the noradrenergic neuron area was also found in the ovarian stroma (Fig. 5d).

## Discussion

In this study using Long Evans CII-ZV rats, ovarian intrinsic innervation was associated with all functional structures of the ovary, suggesting active participation of these neurons in processes, such as development and loss of function. The neural influence on ovarian functions and dysfunction has been poorly studied. The extrinsic innervation of the ovaries regulates follicular development, steroidogenesis, and ovulation (Doganay et al. 2010), and the nerve fibers reach the theca cells without crossing the basal membrane of the follicles, CL, or cysts (Anesetti et al. 2001; D'Albora et al. 2002, 2000). However, little is known about the intrinsic innervation of the mammalian ovary, especially in senescent animals.

Previous studies (Anesetti et al. 2001; D'Albora et al. 2002, 2000; D'Albora and Barcia 1996; McKey et al. 2019) have shown intrinsic neurons in the mammalian ovary, including the human ovary. In Wistar rats, neuronal somas have been identified, mainly in the ovarian medulla and some isolated neurons around the ovarian follicles. Long Evans CII-ZV rats are a good model for reproductive studies because the 4-day estrous cycle is very regular and senescent process well described. Our results confirm the first assumptions and showed that the neurons are associated with the inner theca cells and basal lamina. The present study contributes that, in both young adult and senescent ovaries, most neurons are associated in clusters in all structures of the ovarian cortex (OS and around follicles, CL, and cysts).

The abundant innervation that each follicle has from early development until cell death suggests involvement in the regulation of the development of follicles and ovulation (Madekurozwa 2008). Significant levels of NE have been observed inside the ovaries of senescent rats, but the concentration of NE in the extrinsic innervation (such as the celiac ganglion) decreases with age (Chavez-Genaro et al. 2007; Cruz et al. 2017). This neurotransmitter could be synthesized by other sources, such as the intrinsic innervation. We demonstrated subpopulations of TH-positive neurons (limiting enzyme to synthesize NE) that co-express NeuN and found an increased neuron size related to increasing age, but this was associated with high production of NE in the senescent ovary.

The increased size of the somas of intrinsic neurons (NeuN-positive and TH-positive) related to ovarian cysts and OS in senescent animals, coupled with a significant decrease in the number of these neurons, allows us to propose an exacerbated cell machine in the maintenance of ovarian cysts and high concentration of NE related to reproductive senescence and various pathologies (Chavez-Genaro et al. 2007; Cruz et al. 2017).

Studies of extrinsic innervation in the ovary have shown that they release neurotransmitters into the ovary; NE, VIP, and Ach, among others, may be responsible for some metabolic activity of the functional structures of the ovary (Dissen et al. 2002; Hsueh et al. 1984; Mayerhofer et al. 1997). For example, follicles growing in highly innervated ovarian regions may have a selective advantage over others that are not exposed to neurotransmitter signals (Mayerhofer et al. 1997). In addition, Ach (Lakomy et al. 1982; Mayerhofer et al. 1997), NE (Masuda et al. 2001), and VIP (Bruno et al. 2011) regulate ovarian steroidogenesis depending on the stage of the estrous cycle.

In the rat, the relationship between intrinsic neurons in the ovary and age, especially during the loss of reproductive function, allows us to suggest that 12 months of age is key to the start of decaying ovarian function in rats. At this point, there is an increase in NE inside the ovary (Acuna et al. 2009; Chavez-Genaro et al. 2007; Cruz et al. 2017), which may be produced by large intrinsic neurons. Several authors have proposed that sympathetic hyperactivity may contribute to the development and progression of polycystic ovarian syndrome (PCOS) (Morales-Ledesma et al. 2010). In young adult animals with PCOS induced by neonatal estradiol valerate administration, there is a high concentration of NE in the ovary, whereas the estradiol level is low. This causes a reduction in follicular growth, which in turn causes ovarian failure, loss of cyclicity, and reduced fertility (Lara et al. 2000; Lara et al. 1990a, b). The senescent rats have several characteristics similar to PCOS in young adult rats, including follicular cysts occupying the major ovarian cortex area, anovulation, high production of androgen, low estradiol levels, and persistent vaginal cornification (Linares et al. 2019). These effects are also similar to those observed in women with PCOS.

NGF and brain-derived neurotrophic factor (BDNF), which promote neurogenesis and neuronal plasticity in the peripheral nervous system of both developing and adult animals, also function as regulators of follicular development, ovulation, and steroidogenesis (Russo et al. 2012). In animals with PCOS, both factors are increased (Chang et al. 2019; Streiter et al. 2016). However, in postmenopausal women or those with a lower follicular reserve, the levels of NGF and BDNF are decreased (Begliuomini et al. 2007; Pluchino et al. 2009a, b). This could be related to a decreased number of neurons in the gonads of senescent animals, as BNDF significantly decreases after the animal stops cycling (approximately 12 months).

Estrogen receptors are present in the sympathetic, parasympathetic, and sensorial neurons that innervate the ovaries of adult animals. Long-term treatment with 17β-estradiol reduces the populations of these neurons (Jana et al. 2012, 2013; Koszykowska et al. 2011). It is possible that, in rats, the intrinsic innervation activity in early and adult stages may be regulated by ovarian steroids in addition to the extrinsic innervation (Acuna et al. 2009).

The neuronal markers NeuN and β-tubulin have been used to describe the intrinsic innervation of the ovaries of neonatal, juvenile and young adult rats, as well as other mammalian species, including the human ovary (Anesetti et al. 2001). In the present work we did not find differences in the kind of neurons immunolabeling to NeuN and β-tubulin, that is, the same neuron displayed reactivity to both. In the intrinsic neurons of the ovary, we did not find differences between both neuronal markers related to age, as has been described for other organs (Baas et al. 2016; Jiang and Oblinger 1992; Korzhevskii et al. 2011).

## Conclusions

In the ovaries of CII-ZV rats, the intrinsic neurons are in the follicular theca, around the CL, in the OS, and around cysts. These results suggest active participation of these neurons in processes of development and remodeling of ovarian structures. As the morphology and number of these neurons vary with age, especially in senescent rats, we suggest that the intrinsic neurons are a part of the age-related loss of ovarian functions.

## Data Availability

The data sets generated and/or analyzed during the current study are available from the corresponding author upon reasonable request.

## Abbreviations

CL: Corpus luteum
NE: Norepinephrine
NGF: Nerve growth factor
OS: Ovarian stroma
Ach: Acetylcholinesterase
PCOS: Polycystic ovarian syndrome
BDNF: Brain-derived neurotrophic factor
NeuN: Neuron-specific nuclear protein
TH: Tyrosine hydroxylase
VIP: Vasoactive intestinal peptide

## References

[CR1] Acuna E, Fornes R, Fernandois D, Garrido MP, Greiner M, Lara HE, Paredes AH (2009). Increases in norepinephrine release and ovarian cyst formation during ageing in the rat. Reprod Biol Endocrinol.

[CR2] Aguado LI (2002). Role of the central and peripheral nervous system in the ovarian function. Microsc Res Tech.

[CR3] Almeida-Souza L, Timmerman V, Janssens S (2011). Microtubule dynamics in the peripheral nervous system: a matter of balance. BioArchitecture.

[CR4] Anesetti G, Lombide P, D'Albora H, Ojeda SR (2001). Intrinsic neurons in the human ovary. Cell Tissue Res.

[CR5] Anetsberger D, Kurten S, Jabari S, Brehmer A (2018). Morphological and immunohistochemical characterization of human intrinsic gastric neurons. Cells Tissues Organs.

[CR6] Baas PW, Rao AN, Matamoros AJ, Leo L (2016). Stability properties of neuronal microtubules. Cytoskeleton (hoboken).

[CR7] Baljet B, Drukker J (1979). The extrinsic innervation of the abdominal organs in the female rat. Acta Anat (basel).

[CR8] Banerjee S, Banerjee S, Saraswat G, Bandyopadhyay SA, Kabir SN (2014). Female reproductive aging is master-planned at the level of ovary. PLoS ONE.

[CR9] Begliuomini S, Casarosa E, Pluchino N, Lenzi E, Centofanti M, Freschi L (2007). Influence of endogenous and exogenous sex hormones on plasma brain-derived neurotrophic factor. Hum Reprod.

[CR10] Block E (1951). Quantitative morphological investigations of the follicular system in women; methods of quantitative determinations. Acta Anat (basel).

[CR11] Block E (1952). Quantitative morphological investigations of the follicular system in women; variations at different ages. Acta Anat (basel).

[CR12] Bruno JB, Matos MHT, Chaves RN, Figueiredo JRD (2011). Involvement of vasoactive intestinal peptide (VIP) on ovarian physiology. Anim Reprod.

[CR13] Chang HM, Wu HC, Sun ZG, Lian F, Leung PCK (2019). Neurotrophins and glial cell line-derived neurotrophic factor in the ovary: physiological and pathophysiological implications. Hum Reprod Update.

[CR14] Chavez-Genaro R, Lombide P, Dominguez R, Rosas P, Vazquez-Cuevas F (2007). Sympathetic pharmacological denervation in ageing rats: effects on ovulatory response and follicular population. Reprod Fertil Dev.

[CR15] Cruz G, Fernandois D, Paredes AH (2017). Ovarian function and reproductive senescence in the rat: role of ovarian sympathetic innervation. Reproduction.

[CR16] D'Albora H, Anesetti G, Lombide P, Dees WL, Ojeda SR (2002). Intrinsic neurons in the mammalian ovary. Microsc Res Tech.

[CR17] D'Albora H, Barcia JJ (1996). Intrinsic neuronal cell bodies in the rat ovary. Neurosci Lett.

[CR18] D'Albora H, Lombide P, Ojeda SR (2000). Intrinsic neurons in the rat ovary: an immunohistochemical study. Cell Tissue Res.

[CR19] Dissen GA, Romero C, Paredes A, Ojeda SR (2002). Neurotrophic control of ovarian development. Microsc Res Tech.

[CR20] Doganay M, Simsek A, Tapisiz OL, Mulazimoglu BS, Yumusak N, Gungor T (2010). Superior ovarian nerve (SON) transection leads to stunted follicular maturation: a histomorphologic and morphometric analysis in the rat model. Fertil Steril.

[CR21] Dredge BK, Jensen KB (2011). NeuN/Rbfox3 nuclear and cytoplasmic isoforms differentially regulate alternative splicing and nonsense-mediated decay of Rbfox2. PLoS ONE.

[CR22] Duan W, Zhang YP, Hou Z, Huang C, Zhu H, Zhang CQ, Yin Q (2016). Novel insights into NeuN: from neuronal marker to splicing regulator. Mol Neurobiol.

[CR23] Dumesic DA, Richards JS (2013). Ontogeny of the ovary in polycystic ovary syndrome. Fertil Steril.

[CR24] Gilbert AB (1969). Innervation of the ovary of the domestic hen. Q J Exp Physiol Cogn Med Sci.

[CR25] Guselnikova VV, Korzhevskiy DE (2015). NeuN as a neuronal nuclear antigen and neuron differentiation marker. Acta Nat.

[CR26] Hao MM, Fung C, Boesmans W, Lowette K, Tack J, Vanden Berghe P (2020). Development of the intrinsic innervation of the small bowel mucosa and villi. Am J Physiol Gastrointest Liver Physiol.

[CR27] Hsueh AJ, Adashi EY, Jones PB, Welsh TH (1984). Hormonal regulation of the differentiation of cultured ovarian granulosa cells. Endocr Rev.

[CR28] Jana B, Lata M, Bulc M, Calka J (2012). Long term estradiol-17beta administration changes population of the dorsal root ganglia neurons innervating the ovary in the sexually mature gilts. Neuropeptides.

[CR29] Jana B, Rytel L, Czarzasta J, Calka J (2013). Reduction of the number of neurones in the caudal mesenteric ganglion innervating the ovary in sexually mature gilts following testosterone administration. J Neuroendocrinol.

[CR30] Jana B, Calka J, Rytel L, Czarzasta J (2015). Morphological and neurochemical characterization of the ovarian sympathetic chain ganglia perikarya in testosterone-treated sexually matured pigs. Ann Anat.

[CR31] Jana B, Meller KA, Czajkowska M, Calka J (2018). Long-term estradiol-17beta exposure decreases the cholinergic innervation pattern of the pig ovary. Ann Anat.

[CR32] Jiang YQ, Oblinger MM (1992). Differential regulation of beta III and other tubulin genes during peripheral and central neuron development. J Cell Sci.

[CR33] Katsetos CD, Legido A, Perentes E, Mork SJ (2003). Class III beta-tubulin isotype: a key cytoskeletal protein at the crossroads of developmental neurobiology and tumor neuropathology. J Child Neurol.

[CR34] Kim KK, Adelstein RS, Kawamoto S (2009). Identification of neuronal nuclei (NeuN) as Fox-3, a new member of the Fox-1 gene family of splicing factors. J Biol Chem.

[CR35] Korzhevskii DE, Karpenko MN, Kirik OV (2011). Microtubule-associated proteins as markers of nerve cell differentiation and functional status. Morfologiia.

[CR36] Koszykowska M, Calka J, Szwajca P, Jana B (2011). Long-term estradiol-17beta administration decreases the number of neurons in the caudal mesenteric ganglion innervating the ovary in sexually mature gilts. J Reprod Dev.

[CR37] Lakomy M, Doboszynska T, Szteyn S (1982). Cholinergic nerves in the ovary, the uterine tube and the uterus in pig. Folia Morphol (warsz).

[CR38] Lara HE, McDonald JK, Ahmed CE, Ojeda SR (1990). Guanethidine-mediated destruction of ovarian sympathetic nerves disrupts ovarian development and function in rats. Endocrinology.

[CR39] Lara HE, McDonald JK, Ojeda SR (1990). Involvement of nerve growth factor in female sexual development. Endocrinology.

[CR40] Lara HE, Dissen GA, Leyton V, Paredes A, Fuenzalida H, Fiedler JL, Ojeda SR (2000). An increased intraovarian synthesis of nerve growth factor and its low affinity receptor is a principal component of steroid-induced polycystic ovary in the rat*. Endocrinology.

[CR41] Linares R, Rosas G, Vieyra E, Ramirez DA, Velazquez DR, Espinoza JA (2019). In adult rats with polycystic ovarian syndrome, unilateral or bilateral vagotomy modifies the noradrenergic concentration in the ovaries and the celiac superior mesenteric ganglia in different ways. Front Physiol.

[CR42] Lind D, Franken S, Kappler J, Jankowski J, Schilling K (2005). Characterization of the neuronal marker NeuN as a multiply phosphorylated antigen with discrete subcellular localization. J Neurosci Res.

[CR43] Madekurozwa MC (2008). An immunohistochemical study of ovarian innervation in the emu (Dromaius novaehollandiae). Onderstepoort J Vet Res.

[CR44] Mariani M, Karki R, Spennato M, Pandya D, He S, Andreoli M (2015). Class III beta-tubulin in normal and cancer tissues. Gene.

[CR45] Masuda M, Kubota T, Aso T (2001). Effects of nitric oxide on steroidogenesis in porcine granulosa cells during different stages of follicular development. Eur J Endocrinol.

[CR46] Mayerhofer A, Dissen GA, Costa ME, Ojeda SR (1997). A role for neurotransmitters in early follicular development: induction of functional follicle-stimulating hormone receptors in newly formed follicles of the rat ovary. Endocrinology.

[CR47] Mayerhofer A, Smith GD, Danilchik M, Levine JE, Wolf DP, Dissen GA, Ojeda SR (1998). Oocytes are a source of catecholamines in the primate ovary: evidence for a cell-cell regulatory loop. Proc Natl Acad Sci USA.

[CR48] McKey J, Bunce C, Batchvarov IS, Ornitz DM, Capel B (2019). Neural crest-derived neurons invade the ovary but not the testis during mouse gonad development. Proc Natl Acad Sci USA.

[CR49] Morales-Ledesma L, Linares R, Rosas G, Moran C, Chavira R, Cardenas M, Dominguez R (2010). Unilateral sectioning of the superior ovarian nerve of rats with polycystic ovarian syndrome restores ovulation in the innervated ovary. Reprod Biol Endocrinol.

[CR50] Mullen RJ, Buck CR, Smith AM (1992). NeuN, a neuronal specific nuclear protein in vertebrates. Development.

[CR51] Pastelin CF, Rosas NH, Morales-Ledesma L, Linares R, Dominguez R, Moran C (2017). Anatomical organization and neural pathways of the ovarian plexus nerve in rats. J Ovarian Res.

[CR52] Peng MT, Huang HH (1972). Aging of hypothalamic-pituitary-ovarian function in the rat. Fertil Steril.

[CR53] Pluchino N, Cubeddu A, Begliuomini S, Merlini S, Giannini A, Bucci F (2009). Daily variation of brain-derived neurotrophic factor and cortisol in women with normal menstrual cycles, undergoing oral contraception and in postmenopause. Hum Reprod.

[CR54] Pluchino N, Cubeddu A, Giannini A, Merlini S, Cela V, Angioni S, Genazzani AR (2009). Progestogens and brain: an update. Maturitas.

[CR55] Portiansky EL, Barbeito CG, Gimeno EJ, Zuccolilli GO, Goya RG (2006). Loss of NeuN immunoreactivity in rat spinal cord neurons during aging. Exp Neurol.

[CR56] Russo N, Russo M, Daino D, Bucci F, Pluchino N, Casarosa E (2012). Polycystic ovary syndrome: brain-derived neurotrophic factor (BDNF) plasma and follicular fluid levels. Gynecol Endocrinol.

[CR57] Streiter S, Fisch B, Sabbah B, Ao A, Abir R (2016). The importance of neuronal growth factors in the ovary. Mol Hum Reprod.

[CR58] te Velde ER, Dorland M, Broekmans FJ (1998). Age at menopause as a marker of reproductive ageing. Maturitas.

[CR59] Van Nassauw L, Wu M, De Jonge F, Adriaensen D, Timmermans JP (2005). Cytoplasmic, but not nuclear, expression of the neuronal nuclei (NeuN) antibody is an exclusive feature of Dogiel type II neurons in the guinea-pig gastrointestinal tract. Histochem Cell Biol.

[CR60] Venegas-Meneses B, Padilla JF, Juarez CE, Moran JL, Moran C, Rosas-Murrieta NH (2015). Effects of ovarian dopaminergic receptors on ovulation. Endocrine.

[CR61] Weyer A, Schilling K (2003). Developmental and cell type-specific expression of the neuronal marker NeuN in the murine cerebellum. J Neurosci Res.

